# Evaluation of neutrophil gelatinase-associated lipocalin (NGAL) levels as a biomarker for the early diagnosis of heart failure patients without of kidney disease

**DOI:** 10.34172/jcvtr.025.33007

**Published:** 2025-06-28

**Authors:** Anahita Asadolahi Mashhadian, Hashem Nayeri, Ziba Rezvani Sichani

**Affiliations:** Department of Biochemistry, Falavarjan Branch, Islamic Azad University, Isfahan, Iran

**Keywords:** Neutrophil Gelatinase-Associated Lipocalin, Heart failure, C-reactive protein, Creatinine, Cardiac troponin I

## Abstract

**Introduction:**

Neutrophil Gelatinase-Associated Lipocalin (NGAL) is a specific early diagnostic biomarker for acute kidney injury and has shown high diagnostic value across various types of injuries with different etiologies. However, its role in heart failure (HF) diagnosis remains under investigation. This study aims to assess NGAL levels as a potential biomarker for the early detection of HF in patients without of kidney disease.

**Methods:**

A total of 118 participants were enrolled from Shahid Ashrafi and Saei Khomeini Hospitals, including 59 patients with HF and 59 healthy controls. The patients was 48 years, while the healthy controls had an average age of 46 years. The patient group was diagnosed with heart failure with reduced ejection fraction (HFREF, EF<40%) and had no history of kidney disease. After providing written informed consent, they were enrolled in the study: (code IR.IAU. FALA.REC.1397.024). Blood samples were collected from all participants to measure BUN (Blood Urea Nitrogen), creatinine, cardiac troponin I (CTNI), C-reactive protein (CRP), NGAL, and white blood cell (WBC) count.

**Results:**

The results revealed significantly higher serum levels of NGAL, CRP, CTNI, CR, and BUN in the patient group compared to healthy controls. A significant relationship was found between these biomarkers and the incidence of HF in individuals without prior kidney disease (*P* value<0.001).

**Conclusion:**

In conclusion, NGAL can be used to accurately predict the presence of HF without a history of kidney disease of cases, suggesting its potential as an early diagnostic tool for HF in such patients.

## Introduction

 Heart failure (HF) is a major cause of morbidity and mortality worldwide, significantly impacting both public health and healthcare system.^1^ Epidemiological studies have shown that in approximately half of the patients with heart failure (HF), the ejection fraction (EF) remains within the normal range ( ≥ 40-50%). patients with HF are currently classified into one of these two groups: First; HF with reduced EF (commonly referred to as systolic heart failure) and Second: HF with preserved EF (referred to as diastolic heart failure).^2^ Many patients with heart failure suffer from varying degrees of renal dysfunction. Renal impairment is defined by an increase in blood urea nitrogen (BUN) levels exceeding 30 mg/dL and serum creatinine levels greater than 1.5 mg/Dl.^3^ Studies show that 57% of patients with heart failure had serum creatinine levels above 1.5 mg/Dl.^4^ Additionally, among eight thousand heart failure patients, 29% had moderate to severe kidney failure, while 63% had mild kidney dysfunction. Kidney dysfunction exacerbates and accelerates atherosclerosis, ventricular hypertrophy, and cardiac remodeling.^5^ As heart function worsens, the insufficient blood supply to the kidneys, due to a reduction in the left ventricular ejection fraction, further progresses kidney dysfunction.^6^ This leads to a decreased response to diuretics, diuretic resistance, and an increase in the body’s fluid load, which negatively impacts heart function.^6^ Complete blood cell count, electrolyte measurements, blood urea nitrogen, serum creatinine, liver enzymes, and comprehensive urinalysis are the necessary tests conducted in newly diagnosed HF patients as well as in patients with chronic HF experiencing acute exacerbation.^7^ It is characterized by the heart’s inability to pump blood efficiently, leading to inadequate perfusion of tissues and organs.^8^ Despite advances in diagnosis and treatment, early detection and management of HF remain challenging, particularly in the absence of clear symptoms in the early stages.^9^ Consequently, the demand for innovative biomarkers remains critical to enhance early detection, refine prognostic assessments, and inform therapeutic approaches in patients with heart failure (HF).^8,9^ Among these emerging biomarkers, Neutrophil Gelatinase-Associated Lipocalin (NGAL) has garnered significant interest as a promising candidate.^10^ NGAL is a 25 kDa glycoprotein, primarily produced by neutrophils and epithelial cells, which has been identified as a sensitive marker for acute kidney injury (AKI). It is rapidly upregulated in response to renal damage and has shown great promise as an early diagnostic biomarker in the context of AKI.^11^ Beyond its role in renal injury, emerging evidence suggests that NGAL may also be involved in various other pathological conditions, including cardiovascular diseases. Its presence and elevated levels have been linked to inflammation, oxidative stress, and tissue injury, all of which are central to the pathophysiology of heart failure.^12^ While there is substantial research exploring the role of NGAL in kidney disease, its utility in diagnosing heart failure—particularly in patients without a history of kidney disease—remains underexplored.^13^ Troponins are a family of proteins found in skeletal and cardiac muscle fibers that facilitate muscle contraction.^14^ Troponin testing measures the level of specific cardiac troponins in the blood to aid in the diagnosis of heart injuries. HF can occur with or without concurrent kidney dysfunction, and the presence of renal impairment in HF patients often complicates the diagnosis and management of both conditions.^15^ In this context, a biomarker like NGAL, which is sensitive to both renal and cardiovascular stresses, could provide valuable insights into the early stages of heart failure, even in the absence of pre-existing kidney disease.^16^ Several studies have suggested that NGAL levels are elevated in patients with chronic heart failure, but its diagnostic value in patients without a history of kidney disease has not been thoroughly investigated.^17^ Additionally, there is limited data on whether NGAL levels correlate with the severity or progression of HF in such patients. Understanding the potential of NGAL as a diagnostic tool could not only enhance early detection but also improve the management of heart failure, especially in its early stages, when intervention can significantly alter the disease course.^18^ This study aims to evaluate NGAL levels as a biomarker for the early diagnosis of heart failure in patients who do not have of kidney disease. By assessing serum NGAL levels in this cohort, we seek to determine whether NGAL can serve as a reliable indicator of heart failure, providing a valuable tool for clinicians in identifying at-risk individuals.

## Materials and Methods

###  Serum preparation

 In the present study, a total of 118 participants were from among the visitors to Shahid Ashrafi and Sai Khomeini Hospitals. this study is a cross-sectional study that consisting of 59 patients and 59 healthy controls. According to statistical analysis, the average age of the patients was 48 years, while the average age of the healthy controls was 46 years. Overall, 51% of the participants were women and 47% were men. In the initial phase, 59 patients with HF and without a history of kidney disease were identified. These patients were classified as HFREF, which refers to heart failure with reduced ejection fraction (EF < 40%), a condition in which the heart’s contraction is impaired, leading to a decrease in ejection fraction.^19^ Subsequently, 59 healthy individuals without a history of hereditary diseases and who were not on any medication were selected. After providing written informed consent, they were enrolled in the study: (code; IR.IAU.FALA.REC.1397.024), and Individuals with a history of kidney disease were excluded from the study. Then, Blood samples of 5 milliliters were collected from the participants after an overnight fast of 8 to 24 hours.

###  Biochemical Analysis

 After blood samples were collected, the CBC samples were rotated on a rotator for several minutes to ensure thorough mixing. They were then immediately processed within the first hour using the Sysmex analyzer, and the white blood cell count was recorded. For the coagulated samples, efforts were made to complete the centrifugation process (centrifugation at 3000 rpm for approximately 5 to 10 minutes) and serum separation, and store the samples in the freezer within one hour of collection. This procedure was followed to prevent any potential errors regarding the stability of the parameters. BUN, Creatinine, and CRP tests were conducted on the same day of collection using the Mindray auto analyzer,^20^ while Troponin levels were measured with the Widex analyzer.^21^ A portion of the serum was stored in the freezer for NGAL measurement.

###  Measurement of NGAL Level

 This kit is based on the sandwich enzyme-linked immunosorbent assay (ELISA) method and employs monoclonal antibodies. It is the most common ELISA method where an antigen is captured between two specific antibodies. One antibody is coated on the wells to capture the antigen, and the second antibody, conjugated with an enzyme, acts as a detector. In this method, wells are coated with antibodies against a specific NGAL antigen. Patient samples, covered with antibody-coated wells, are added adjacent to these wells and are captured by the coated antibodies. Then, Biotinylated NGAL, acting as a detector, is added. After incubation and washing, Horseradish Peroxidase (HRP)-Conjugated Avidin, which conjugates with the second antibody, is added to the wells and incubated. The amount of the immune complex formed in the wells is proportional to the NGAL concentration in the samples. After washing, a chromogenic solution (TMB), containing hydrogen peroxide and chromogen, is added to the wells. The blue color formed is proportional to the immune complex formed in the wells. By adding the stop solution, the blue color changes to yellow, which has the best optical absorption at a wavelength of 450 nanometers during measurement.

###  Statistical Analysis

 In this study, independent samples t-test and chi-square test were employed for statistical analysis. The data were assumed to follow a normal distribution. The analyses were conducted using SPSS version 20 software.

## Results

###  Correlation Coefficients Between Serum NGAL Levels with CTNI and CRP

 In this study, no significant correlations were observed between serum NGAL levels with serum troponin levels, and serum CRP levels, in either the patient or the healthy group. These findings suggest that NGAL may not be directly associated with troponin or CRP levels in the context of heart failure or in healthy individuals (Table 1).

**Table 1:** Pearson Correlation Coefficients Between Serum NGAL Levels with CTNI and CRP

** Variable**	** Group**	** Serum NGAL Level**	
		** r**	***P***
CTNI ng/ml CRP mg/L	Patient Healthy Patient Healthy	0.168 -0.043 -0.013 0.140	0.20 0.75 0.92 0.29

Abbreviations: CTN1; Cadiac troponin I, CRP; C-reactive protein, r; Pearson correlation coefficient, *P*; *P* value

###  Comparison of Biochemical parameters Between Two Groups: Patients and Healthy Controls

 This study revealed that individuals with heart failure had significantly elevated creatinine levels compared to healthy controls. Renal parameters, such as BUN and creatinine (Cr), were analyzed, and the findings indicated a significant association between serum urea and creatinine levels and the occurrence of heart failure (HF) in patients without prior kidney disease (*P *value < 0.001). The glomerular filtration rate (GFR) was calculated using the MDRD formula, which incorporates variables such as age, gender, and blood creatinine levels.^22^ This formula is specifically designed for individuals aged 18 years and older. Based on the standard range, a GFR exceeding 59 suggests normal kidney function. In both the patient and control groups, the GFR values were above 59. The results also demonstrated that serum troponin levels were markedly higher in the patient group compared to the healthy group, establishing a significant correlation between elevated serum troponin and the prevalence of HF in individuals without kidney disease (*P *value < 0.001). Similarly, the mean serum CRP levels were notably higher in patients than in healthy individuals, indicating a significant relationship between increased CRP levels and the incidence of HF in those without a history of kidney disease (*P* value < 0.001). However, no significant difference was observed in the mean WBC levels between the two groups. This lack of correlation could be attributed to the early stages of heart failure in the patients, during which the inflammatory response and leukocyte elevation may not yet be prominent. Additionally, serum NGAL levels were significantly higher in patients with heart failure compared to healthy controls, with the difference being statistically significant (Table 2).

**Table 2:** Mean Serum Biochemical parameters in Two Groups

**Variable**	**Group**	**Mean**	**SD**	**Independent t-test **	
				**t-value **	***P*** ** value**
BMI kg/m	Patient	22/97	2/30		
	Healthy	22/20	2/ 20	1.84	0.07
GFR ml/min	Patient	60/20	17/94		
	Healthy	62/22	16/14	0.82	0.48
CTNI ng/ml	Patient	2.39	1.07		
	Healthy	1.41	0.94	5.30	< 0.001
NGLA ng/ml	Patient	236.18	92.72		
	Healthy	152.46	57.53	5.89	< 0.001
WBC cell/ml	Patient	7592.03	2413.67		
	Healthy	7027.12	1746.51	1.46	0.15
Age Years	Patient	49	12/22		
	Healthy	47	11/26	1.01	0.31
CRP mg/L	Patient	7.32	5. 24		
	Healthy	3.27	1.36	5.74	< 0.001
Cr mg/dL	Patient	1.22	0.95		
	Healthy	0.23	2.20	4.99	< 0.001
Urea mg/dL	Patient	23.21	7.95		
	Healthy	14.14	3.62	7.98	< 0.001

Abbreviations: BMI; Body Mass Index, GFR; Glomerular Filtration Rate, Cr; Creatinine,WBC; White Blood Cell,SD; Standard Deviation

###  Logistic Regression Analysis for Predicting Non-Chronic Kidney Disease HF Based on NGAL, Urea, Creatinine, Troponin, and CRP Values

 Overall, using the values of NGAL, urea, creatinine, troponin, and CRP, it is possible to accurately predict the presence of heart failure (HF) without a history of kidney disease of cases. According to the Wald values and P-values provided in Table 3, the logistic regression analysis showed that urea, troponin, CRP, and NGAL levels were significant predictors for the development of HF without a history of kidney disease.

**Table 3:** Logistic Regression Analysis for Predicting Non-Chronic Kidney Disease HF Based on NGAL, Urea, Creatinine, Troponin, and CRP Values

**Variable**	**Beta**	**Number Wald**	***P*** ** value**	**OR**	**OR %confidence interval**	
					**lower bound **	**Upper bound **
CRP mg/L	0.687	9.454	0.002	1.987	1.283	3.079
NGAL ng/ml	0.021	9.179	0.002	1.021	1.007	1.035
Urea mg/dL	0.477	13.286	< 0.001	1.611	1.247	2.081
Creatinine mg/dL	3.033	1.976	0.16	20.769	0.302	1426.701
Troponin ng/ml	1.401	10.145	0.001	4.060	1.714	9.616

Abbreviations: OR; Odds Ratio

## Discussion

 In this study, we investigated various biomarkers to explore their potential relationship with heart failure (HF) in individuals without a history of kidney disease, with a particular focus on Neutrophil Gelatinase-Associated Lipocalin (NGAL).^23^ NGAL, a small protein predominantly expressed on the surface of neutrophils and renal tubular epithelial cells, has been shown to be an early marker of kidney injury and inflammation.^24^ While numerous biomarkers such as creatinine, BUN, troponin, CRP, and WBC have been explored in the context of heart failure, this study highlights the significant association between serum NGAL levels and the incidence of HF, particularly in individuals without prior kidney disease. Our results revealed that serum NGAL levels were significantly higher in individuals with heart failure compared to healthy controls, indicating its potential as a valuable biomarker for assessing the occurrence of HF, even in the absence of established renal dysfunction.^25^ This finding is consistent with previous studies which have reported elevated NGAL levels in patients with heart failure, suggesting its role in the inflammatory response and as an indicator of kidney involvement.^26^ In fact, NGAL is known to be a key mediator in the acute phase response, primarily reflecting renal tubular injury, but it may also be influenced by systemic inflammation, as seen in heart failure.^27^ While other renal biomarkers such as creatinine and BUN have traditionally been used to assess kidney function, NGAL offers several advantages, particularly its early detection of kidney injury before significant changes in creatinine or GFR occur.^28^ In this study, we also observed that the levels of creatinine and BUN were significantly higher in the patient group compared to the healthy group, which aligns with findings from other studies.^29^ However, unlike NGAL, which showed a clear distinction between the two groups, creatinine and BUN levels did not provide as specific a marker for heart failure without prior kidney disease. This suggests that NGAL may be a more sensitive and early marker for identifying heart failure, even in the absence of noticeable renal dysfunction. Interestingly, no significant correlation was observed between serum NGAL levels and other markers, such as troponin or CRP, in either the patient or control group. This lack of correlation may suggest that NGAL operates through different physiological pathways, primarily reflecting renal tubular injury, whereas troponin and CRP are more directly involved in cardiac injury and systemic inflammation.^30^ The absence of a significant correlation between NGAL and troponin in this study aligns with findings from some previous research, which suggests that NGAL is a distinct biomarker of renal involvement and may not directly correlate with markers of cardiac injury.^11^ Additionally, the study did not find a significant correlation between NGAL and WBC levels, which may be explained by the fact that the patients were in the early stages of heart failure. In these early stages, systemic inflammation and leukocyte activation may not yet be sufficiently pronounced to correlate with elevated NGAL levels, which are more reflective of ongoing renal stress. The study results indicate that the lower-than-expected creatinine levels in the healthy group may be due to differences in hydration status, muscle mass, and physiological variations between the healthy individuals and heart failure patients without kidney disease. Moreover, heart failure patients, even without kidney disease, may experience renal hypoperfusion (reduced renal blood flow), which can affect creatinine metabolism. These patients may have higher creatinine levels compared to healthy individuals, even if their kidney function is normal, which can further accentuate the differences between the groups.

 The study results indicate that the lower-than-expected creatinine levels in the healthy group may be due to differences in hydration status, muscle mass, and physiological variations between the healthy individuals and heart failure patients without kidney disease. Moreover, heart failure patients, even without kidney disease, may experience renal hypoperfusion (reduced renal blood flow), which can affect creatinine metabolism. These patients may have higher creatinine levels compared to healthy individuals, even if their kidney function is normal, which can further accentuate the differences between the groups.

## Conclusion

 In conclusion, NGAL emerges as a promising biomarker for heart failure, particularly in patients without prior kidney disease. Its early detection of kidney injury and association with heart failure underscores its potential role in clinical settings as a tool for early diagnosis and monitoring of heart failure, even before more significant changes in renal function are apparent. Future studies should aim to explore the mechanistic pathways through which NGAL contributes to the progression of heart failure and assess its utility in larger, diverse populations.

## Competing Interests

 The authors declare that there are no conflicts of interest.

## Ethical Approval

 The Ethics Committee of Falavarjan University approved the current study (IR.IAU.FALA.REC.1397.024).
